# Office-Based Arthroscopy Versus Arthrocentesis as Treatment for Temporomandibular Joint Pain and Dysfunction: Preliminary Results of a Randomized Controlled Trial

**DOI:** 10.3390/jcm14092929

**Published:** 2025-04-24

**Authors:** Yang Hang Tang, Nico B. van Bakelen, Barzi Gareb, Fred K. L. Spijkervet

**Affiliations:** Department of Oral and Maxillofacial Surgery, University Medical Center Groningen, University of Groningen, 9700RB Groningen, The Netherlands; b.gareb@umcg.nl (B.G.); f.k.l.spijkervet@umcg.nl (F.K.L.S.)

**Keywords:** therapeutic irrigation, temporomandibular joint disc, osteoarthritis, joint diseases, pain, minimally invasive surgical procedures, craniomandibular disorders, jaw

## Abstract

**Background/Objectives**: Arthroscopy and arthrocentesis are routinely performed for temporomandibular joint (TMJ) disorders, but high-quality evidence regarding their efficacy relative to each other is scarce. The current study, as part of an ongoing randomized controlled trial, aimed to compare office-based arthroscopic lysis and lavage with arthrocentesis for TMJ pain and dysfunction. **Methods**: Adults (≥18 years old) referred to a tertiary care hospital with TMJ arthralgia were included. The exclusion criteria comprised systemic rheumatic disease, connective tissue disease, bony ankylosis, congenital or acquired dentofacial deformities, a history of significant jaw trauma, or systemic illnesses. The primary outcome was joint pain during mandibular movement/function (visual analog scale (VAS); 0–100 mm). The secondary outcomes included pain at rest (VAS), maximum mouth opening (mm), maximum mouth opening without increased pain (mm), protrusive and lateral movements (mm), joint noises (absent/present), and mandibular function (mandibular function impairment questionnaire score). The outcomes were registered at baseline and 3-, 6-, and 12-month follow-ups. Linear mixed models and mixed-effects logistic regressions were utilized to evaluate the effects of interventions on the repeated outcome measurements. **Results**: Twenty subjects were randomly allocated to office-based arthroscopic lysis and lavage (*n* = 10) or arthrocentesis (*n* = 10). Multivariable mixed-effects models showed significantly higher pain scores during mandibular movement/function in the arthrocentesis group compared with arthroscopy (22.42 mm (95% CI: 5.28 to 39.57); *p* = 0.011). The secondary outcomes were not significantly different between the interventions. **Conclusions**: The preliminary results show the superiority of office-based arthroscopy over arthrocentesis in reducing pain during mandibular movement/function over a follow-up period of 1 year while showing no differences between interventions regarding other study outcomes.

## 1. Introduction

Minimally invasive surgical therapies, such as arthroscopy and arthrocentesis, are frequently indicated for symptomatic temporomandibular joint (TMJ) disorders when conservative treatments fail to provide sufficient symptom relief [1]. Both procedures have demonstrated high effectiveness in symptom (i.e., pain and dysfunction) reduction, with reported success rates of approximately 80–90% [2,3,4,5].

Technological advancements have led to the differentiation of complexity and the possibility of reducing the invasiveness of arthroscopy. The successful development of office-based arthroscopy [6,7] under local anesthesia and arthrocentesis [8] has reduced the need for general anesthesia in initial surgical interventions following unsuccessful conservative treatments for TMJ pain and dysfunction.

Despite their routine use, the choice between arthroscopy and arthrocentesis remains debated and is often dictated by patient-specific factors and surgeon preferences. Arthroscopy offers the advantage of direct joint visualization for diagnostic purposes and the possibility for additional therapeutic maneuvers, while arthrocentesis is often preferred for its simplicity, lower invasiveness, and lower direct costs [3]. Notably, both procedures facilitate the adjuvant use of intra-articular injections, such as hyaluronic acid, which has been suggested to improve joint lubrication and, thus, clinical symptoms [9]. A recent systematic review found no significant difference between arthroscopy and arthrocentesis in reducing pain during mandibular movement, although the evidence was of low to very low quality. However, arthroscopic lysis and lavage was superior to arthrocentesis in maximum mouth opening (MMO) improvement at intermediate-term (6 months to 5 years) follow-up [10]. This review highlighted the lack of high-quality studies directly comparing these interventions. Only a handful of randomized controlled trials have assessed the efficacy of arthroscopic lysis and lavage versus arthrocentesis, with findings showing no significant differences between interventions in pain reduction [11,12]. However, these studies were performed over two decades ago, did not always report results sufficiently, and have a high risk of bias, highlighting the need for high-quality clinical trials to evaluate the efficacy of these treatments for TMJ pain and dysfunction.

The present study is part of an ongoing randomized controlled trial designed to assess the efficacy of office-based, single-portal arthroscopy versus arthrocentesis for the treatment of TMJ pain and dysfunction. Specifically, this study aimed to evaluate post-operative TMJ pain, MMO, protrusive and lateral mandibular movements, joint noises, and patient-perceived mandibular functional impairment over a 1-year follow-up period.

## 2. Materials and Methods

This study is reported according to the CONSORT 2010 statement and was conducted according to the principles of the Declaration of Helsinki (seventh version 2013, Fortaleza, Brazil) and the Dutch ‘Medical Research Involving Human Subjects Act’ (WMO).

### 2.1. Clinical Trial Design

A mono-center, parallel-group randomized controlled trial was performed at the University Medical Center Groningen (UMCG), the Netherlands, a tertiary care hospital. The trial was registered at the International Clinical Trials Registry Platform and the Overview of Medical Research in the Netherlands (NL-OMON20171). A pre-specified protocol was finalized before the trial initiation. Ethical approval was given by the Institutional Review Board of the UMCG (METc 2021/275), and all subjects signed informed consent forms prior to the start of study-related procedures.

### 2.2. Study Population

All study subjects were recruited at the outpatient clinic of the Department of Oral and Maxillofacial Surgery of the UMCG. Patients referred for the management of TMJ disorders were screened for inclusion in this study. The inclusion criteria were subjects of 18 years and older with unilateral symptoms of TMJ arthralgia (or bilateral symptoms in which the non-surgically treated joint had a pain score of <30 mm on a visual analog scale (VAS; scale: 0–100 mm)), proven with a diagnostic intra-articular injection with articaine (4%) and epinephrine (1:100,000) [13], and the presence of TMJ arthralgia after two weeks of non-steroidal anti-inflammatory drugs (NSAIDs) to exclude acute inflammatory pain. The exclusion criteria were subjects suffering from systemic rheumatic diseases, connective tissue disease, bony ankylosis of the TMJ, congenital or acquired dentofacial deformities, a history of jaw trauma that resulted in jaw or joint pain, bony changes or mandibular growth restrictions, a psychiatric disorder, or a medical comorbidity, such as coagulation disorders, diabetes mellitus, kidney failure, cardiac ischemia or failure, or a history of human immunodeficiency virus. Furthermore, subjects were excluded from participating when they had received prior TMJ surgery, were unwilling to participate, did not speak English or Dutch, were pregnant, or used concurrent steroids, sedatives, muscle relaxants, or anti-inflammatory drugs other than the prescribed NSAIDs.

### 2.3. Sample Size Calculation

Prior to the study commencement, an estimation of sample size was performed based on a two-sided α of 0.05, power of 0.80, 3 follow-up measurements, an estimated ρ of 0.5, and an effect size f of 0.41 (based on a 10 mm pain VAS as a clinically relevant difference, as determined by the authors) [14]. The required sample size was estimated to be a total of 140 subjects after accounting for a 10% margin for dropouts. Since no proper effect sizes of office-based arthroscopy are available in the literature, an a priori-determined interim analysis was conducted after the inclusion of 10 subjects per arm, with a final follow-up of 1 year to obtain the most reliable effect sizes for a sample size re-calculation. Data received from unlocking the database for the purpose of sample size recalculation were used for the current study.

### 2.4. Study Procedures

All study subjects were randomly allocated (1:1 ratio) to either the intervention (arthroscopy) or control (arthrocentesis) group by the surgeon using a web-based randomization tool (ALEA clinical, version 18.7, FormsVision BV, Abcoude, The Netherlands) after signing the informed consent forms. Block randomization with random block sizes was performed for the distribution of subjects between the groups. Allocation concealment was ensured due to the central randomization process, thereby preventing the operators from deducing the allocation sequence. The treatment assignment was revealed to the operator immediately prior to the procedures via an email sent by the randomization software, preventing any influence on patient enrollment or baseline assessment. The researcher analyzing the data was blinded to the treatment allocation.

Subjects underwent radiographic assessment using cone-beam computed tomography (CBCT) for intra-operative orientation purposes and evaluation of the extent of pre-treatment bony changes observed. The evaluation of the CBCT images and degenerative joint disease (DJD) diagnoses was based on the classification of the Diagnostic Criteria for Temporomandibular Dysfunction (DC/TMD), as described by Ahmad et al. [15]. The anterior disc displacement (ADD) diagnostic categories were based on patient histories and clinical examinations, as described in the DC/TMD [16].

All procedures were performed under local anesthesia by two experienced TMJ surgeons (FKLS and NBvB). Arthroscopy was performed with a single portal. Prior to the procedure, the TMJ was locally anesthetized with articaine (4%) with epinephrine (1:100,000). Thereafter, an 18-gauge needle was inserted in the posterior recess of the superior joint space, 10–12 mm anterior to the mid-tragus, and 0.9% saline was used to insufflate this joint space. The first needle was replaced by a 1.9 mm diameter cannula with a sharp trocar to enter the joint. Next, the sharp trocar was replaced by a blunt obturator for further joint advancement. A 1.2 mm diameter arthroscope (OnPointTM Scope System; Zimmer Biomet, Warsaw, IN, USA) was then introduced into the cannula to allow the visualization of the articular space. A second 18-gauge needle was then introduced into the joint space as an outflow tract, 7–10 mm anteriorly and 7–10 mm caudally from the first insertion needle. After a diagnostic sweep, the outflow needle was used to cut intra-articular adhesions and/or to inject 0.5 mL of methylprednisolone (40 mg/mL subsynovially) into the inflamed tissue, if present and surgically possible. During the arthroscopy, an attempt was made to lavage the joint with a minimum of 100 mL of 0.9% saline. For the arthrocentesis, two 18-gauge needles were introduced into the upper joint space, in the same locations as during the arthroscopy, after locally anesthetizing the TMJ. Similar to the arthroscopic procedure, an attempt was made to flush a minimum of 100 mL of 0.9% saline through the joint once communication was established. No additional drugs or substances were applied. Following both procedures, a strict soft diet protocol was recommended for six weeks.

### 2.5. Outcome Measures

The primary study outcome was pain during mandibular movement and/or function using the VAS (range: 0–100 mm). The secondary study outcomes included pain at rest using the VAS (range: 0–100 mm), the maximum incisal mouth opening in millimeters using a sliding caliper (MMO; interincisal distance), the MMO without perceiving (increased) pain in millimeters using a sliding caliper, protrusive and lateral mandibular movements in millimeters using a dental probe, joint noises in the last month (present/absent), and the impairment of mandibular function using the validated mandibular function impairment questionnaire (MFIQ; 17 items scored on a Likert scale; score: 0–100) [17].

Outcome measurements were registered at baseline and at each post-operative follow-up control (3, 6, and 12 months) by the operating surgeon and analyzed by a blinded researcher (YHT). MFIQ was registered through digitally sent questionnaires at baseline and each follow-up. In the arthroscopy group, the number of subjects who underwent adhesiolysis and/or subsynovial corticosteroid injections was recorded. Additionally, data were collected for cases where subjects did not receive adhesiolysis or corticosteroid injections, as well as instances where adhesions or synovitis were absent, respectively. Any additional treatment during follow-up was registered.

### 2.6. Statistical Analysis

The baseline characteristic registration included gender, age, diagnosis, the duration of symptoms that were so significant that help from a healthcare provider was sought, the total duration of symptoms, and the lavage volume during treatment.

The results were analyzed using the intention-to-treat principle. Continuous variables with a normal distribution were reported as means and standard deviations (SDs) and compared with the unpaired Student’s *t*-test, whereas non-normally distributed variables were presented as medians with first and third quartiles (Q1–Q3) and compared using the Mann–Whitney U test. The normality of the data was evaluated through visual Q–Q plot inspection and the Shapiro–Wilk test. The categorical variables were expressed as frequencies and percentages and analyzed using Fisher’s exact test. The abovementioned analyses were performed in IBM SPSS statistics for Windows, version 28 (IBM Corp., Armonk, NY, USA).

As primary statistical analyses, linear mixed models and mixed-effects logistic regression were applied to evaluate the effects of the interventions on the repeated outcome measurements. The multivariable models involved fixed effects for the treatment group and the follow-up in days. The study subjects were included as random effects. Additionally, the fixed interactions between the treatment group and time and/or the random effect of time were only included in the model if the multivariable model was significantly improved. Model improvement was tested using likelihood ratio tests. All models with continuous outcomes yielded an estimated regression coefficient (β) with a corresponding 95% confidence interval (95% CI). For outcome measures with dichotomous outcomes, odds ratios with a 95% CI were also calculated. All of the mixed-effects models were performed in R, version 4.0.5 (R Core team; R Foundation for Statistical Computing, Vienna, Austria), using the lme4 package [18]. A *p*-value of ≤0.05 (two-tailed) was considered statistically significant.

## 3. Results

**Figure 1:**
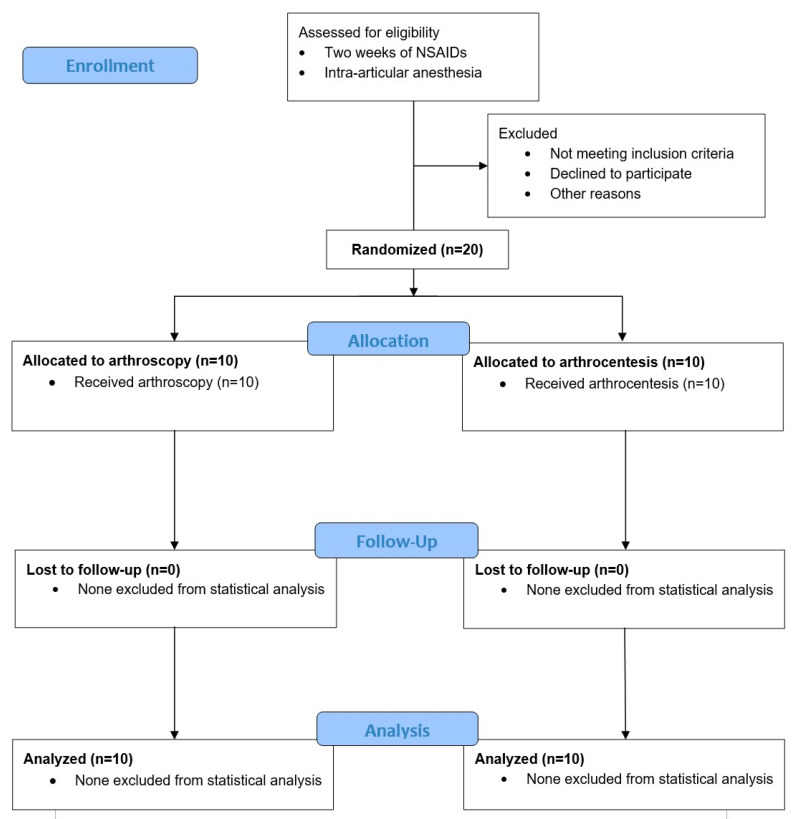
CONSORT flow diagram (version 2010) of subject enrolment, allocation, and follow-up. All subjects allocated to the treatment groups completed follow-up and were included in the analyses.

The first 10 study subjects per study arm of the ongoing trial were enrolled between January 2022 and May 2023 and analyzed after a total follow-up completion time of 1 year. No study subjects were lost to follow-up (Figure 1). In the arthroscopy group, all subjects were females, with a mean age of 45.0 years (17.2). The median duration of symptoms that were so significant that help from a healthcare provider was sought was 4.0 months (2.4–10.5), while the median total duration of symptoms was 15.0 months (3.8–47.3). In the arthrocentesis group, 80% of subjects were female, with a mean age of 36.7 (15.3). The median duration of symptoms that were so significant that help from a healthcare provider was sought was 10.5 months (6.0–15.0), while the median total duration of symptoms was 15.0 months (9.8–48.0) (Table 1). The diagnosis of each study subject and the baseline outcome values are described in Table 1.

In each study arm, additional treatments were performed on three subjects during the follow-up period. In the arthroscopy group, two subjects received corticosteroid injections against the articular capsule, and one subject received arthroscopic surgery under general anesthesia 6 months after the initial treatment. In the arthrocentesis group, all three subjects who underwent an additional treatment received an office-based, single-portal arthroscopy under local anesthesia. During the initial arthroscopy, adhesiolysis was successfully performed in 4 subjects (40%). In 2 subjects (20%), surgical adhesiolysis was not feasible, while 4 subjects (40%) showed no visible intra-articular adhesions. With respect to subsynovial corticosteroid injections, this procedure was successfully carried out in 3 subjects (30%). However, in 6 subjects (60%), the injection could not be performed surgically, and in 1 subject (10%), no synovitis was observed.

The results of each outcome variable at each follow-up are presented in Table 2. The multivariable mixed models show significantly higher pain scores during movement and/or function in the arthrocentesis group compared with arthroscopy (22.42 mm (95% CI: 5.28 to 39.57)) (Table 3). The secondary outcomes were not significantly different between interventions (Table 3). Additionally, a significant improvement over time was observed for the outcomes of pain during movement and/or function, MMO without an increase in pain, MMO, protrusive movements, joint noises, and MFIQ scores, regardless of the treatment employed (Table 3).

One adverse event was registered in the arthroscopy group, which involved the perforation of the external acoustic meatus with the outflow needle. The procedure was stopped immediately after the flushing of saline into the external acoustic meatus was observed. The puncture hole resolved within a few days on its own without any sequelae. Two weeks later, a successful arthroscopy was performed on the same subject.

## 4. Discussion

This study, as part of an ongoing randomized controlled trial, aimed to investigate the efficacy of office-based arthroscopy compared with arthrocentesis for TMJ pain and dysfunction (i.e., painful ADD with/without reduction and DJD). The preliminary results of the trial indicate that arthroscopy was more efficacious in reducing pain during mandibular movement and/or function than arthrocentesis during the 12-month follow-up. No significant differences between the interventions were observed for the secondary outcomes (i.e., pain at rest, MMO without an increase in pain, MMO, protrusive and lateral movements, joint noises, and MFIQ scores).

It has been reported in a previous study that patients with temporomandibular dysfunction may show a large improvement in general health when an absolute pain reduction of 9–19 mm is achieved [19]. Therefore, the magnitude of the pain reduction observed between both treatments over the entire follow-up (22.4 mm on a 0–100 mm VAS) in the current study indicates both a significant and clinically relevant difference. Alternatively, a reduction in the chronic pain VAS score of 10–20% indicates a minimally clinically important difference, while a 30% reduction indicates a moderate difference clinically [20]. Considering the baseline pain scores in the current study of 67.0 mm (Table 1), the 33% difference between groups over the entire follow-up further emphasizes the clinically relevant difference.

A proposed mechanism for pain reduction in both arthrocentesis and arthroscopy is the washing out of pro-inflammatory cytokines and matrix-degrading enzymes responsible for the inflammatory response and subsequent pain [21,22,23]. However, since both arthroscopy and arthrocentesis allow the lavage of the upper joint space, this mechanism alone may not account for the superior effect in pain reduction with arthroscopy. Some studies suggest that factors, such as the lavage volume [21,24] and the duration of symptoms before the initial treatment [25], may influence the treatment outcomes. Alternatively, the superior efficacy of arthroscopy in pain reduction may be partly explained by the subsynovial corticosteroid injections performed during the procedure, which have recently been shown to be an effective maneuver for symptom reduction [26,27]. However, in this study, only three of the ten subjects undergoing arthroscopy received a corticosteroid injection, either because there was no synovitis present or it was surgically unfeasible to perform the injection. The final results will provide further clarification of the relationship in the current trial between the lavage volume, the duration of symptoms, and the subsynovial injection of corticosteroids and pain reduction.

The treatment choice did not appear to have a statistically significant effect on MMO improvement in the current study sample, despite the superiority of arthroscopy in pain reduction and its potential for the targeted lysis of intra-articular adhesions that might restrict mouth opening. These findings indicate that arthrocentesis may be similarly effective in addressing the mechanical intra-articular causes of restricted mouth opening, or that other factors may have played a role, such as secondary muscle contractures after a prolonged period of MMO restriction [28].

A cautious interpretation of the current findings in a clinical context is warranted due to the preliminary nature of this study. Moreover, beyond health outcomes alone, several additional factors are crucial for a comprehensive evaluation of medical interventions. First, economic assessments in relation to potential health benefits are necessary to support clinicians in making optimal treatment choices. The cost-effectiveness of both treatments (based on the primary study outcome and the total costs, and on the MFIQ score and the total costs) will be evaluated and presented after the final study’s completion. Furthermore, the implementation of surgical treatments requires the consideration of the learning curve and the resources required for proper training. For example, performing TMJ arthroscopy requires extensive experience and specialized training to achieve expertise [29], which can limit its widespread adoption. Finally, the treatment choice should consider the additional diagnostic capabilities of arthroscopy that are absent in arthrocentesis. Arthroscopy allows the direct visualization of the internal joint structures, enabling a more accurate understanding of pathological processes and guiding future treatment decisions if the initial surgery is unsuccessful.

A limitation of the current study was the lack of patient blinding, which may have influenced the perception and reporting of subjective patient-reported outcomes, such as pain. However, achieving effective blinding in interventional studies where procedures are performed without general anesthesia presents significant challenges. In the current study, attempts were made to blind the subjects as much as possible by using disposable sterile surgical sheets during the procedure and by using similar operating theatre set-ups. Nevertheless, subjects may have been able to identify their treatment allocation from procedure-specific maneuvers, rendering complete blinding unfeasible. Furthermore, since the current results are based on only a portion of the final sample size, the results are underpowered and may be subject to type-II errors (i.e., false-negative findings). Lastly, the single-center nature of this study, which is being conducted at a tertiary referral center, may limit the generalizability of the findings to the broader population of patients with TMJ disorders. Future studies incorporating a multicenter design would enhance the external validity and, thus, increase the generalizability.

One of the main strengths of the current study lies in its rigorous design, characterized by randomized subject enrolment and researcher blinding. Additionally, the a priori-determined sample size allows this study to be the first one comparing arthroscopy with arthrocentesis that achieves sufficient statistical power. The inclusion of a comprehensive set of patient outcomes allowed a comprehensive interpretation of the clinical effects of the treatments. The use of the MFIQ as a patient-reported outcome measure provided an accurate assessment of each patient’s health status, free from clinician interpretation. Furthermore, mixed-models analysis allowed the longitudinal evaluation of outcomes after both interventions.

Conclusively, these preliminary results suggest a superiority of office-based arthroscopy over arthrocentesis in reducing pain during mandibular movement and/or function for the treatment of TMJ pain and dysfunction after 12 months of follow-up. No differences between the interventions were observed regarding the outcomes of pain at rest, MMO, protrusive and lateral movements, joint noises, and mandibular function. More definitively, better-powered results will be published after the completion of the trial.

## Figures and Tables

**Table 1:** Baseline characteristics.

Characteristics	Arthroscopy	Arthrocentesis
Sample size, *n*	10	10
Female, *n (%)*	10 (100)	8 (80)
Age (years), mean (SD)	45.0 (17.2)	36.7 (15.3)
Duration of symptoms that required healthcare provider assistance (months), median (Q1–Q3)	4.0 (2.4–10.5)	10.5 (6.0–15.0)
Total duration of symptoms (months), median (Q1–Q3)	15.0 (3.8–47.3)	15.0 (9.8–48.0)
ADD, *n* (%)		
No ADD	1 (10)	1 (10)
ADDwR	2 (20)	0 (0)
ADDwR with intermittent locking	2 (20)	1 (10)
ADDwoR with limited mouth opening	4 (40)	8 (80)
ADDwoR without limited mouth opening	1 (10)	0 (0)
DJD, *n* (%)		
No DJD	1 (10)	2 (20)
Indeterminate for DJD	1 (10)	0 (0)
Evidence for DJD	8 (80)	8 (80)
Lavage volume during treatment (ml), mean (SD)	380.0 (71.5)	332.5 (131.3)
Pain during mandibular movement (mm), mean (SD)	67.0 (10.6)	67.0 (16.4)
Pain at rest (mm), median (Q1–Q3)	0.0 (0.0–0.0)	0.0 (0.0–17.5)
MMO without (increase in) pain (mm), mean (SD)	29.6 (6.5)	30.7 (5.2)
MMO (mm), mean (SD)	33.3 (6.5)	36.2 (5.4)
Protrusive movement (mm), mean (SD)	6.2 (2.0)	5.6 (1.7)
Ipsilateral movement (mm), mean (SD)	9.2 (2.2)	7.4 (2.8)
Contralateral movement (mm), mean (SD)	7.2 (2.8)	7.4 (2.7)
MFIQ score, mean (SD)	57.9 (11.8)	61.6 (10.9)
Joint noises present, *n* (%)	5 (50)	6 (60)

Abbreviations: *n*—number of subjects; SD—standard deviation; Q1–Q3—first and third quartiles; ADD—anterior disc displacement; ADDwR—anterior disc displacement with reduction; ADDwoR—anterior disc displacement without reduction; DJD—degenerative joint disease; MMO—maximum mouth opening; MFIQ—mandibular function impairment questionnaire.

**Table 2:** Results of study outcomes registered at each follow-up.

Study Outcome	Follow-up (Months)	Post-Operative Change Scores ^^^		*p*
		Arthroscopy	Arthrocentesis	
Pain during mandibular movement (mm)	3	−25.0 (−15.0 to −62.5) *	0.0 (12.5 to 0.0) *	**0.005**
	6	−35.5 (32.2) ^#^	−6.0 (15.1) ^#^	**0.021**
	12	−47.5 (23.0) ^#^	−21.5 (25.2) ^#^	**0.027**
Pain at rest (mm)	3	0.0 (0.0 to 0.0) *	0.0 (2.5 to −2.5) *	0.796
	6	0.0 (0.0 to 0.0) *	0.0 (10.0 to −10.0) *	0.529
	12	0.0 (0.0 to 0.0) *	0.0 (2.5 to −2.5) *	1.000
MMO without increase in pain (mm)	3	2.1 (3.8) ^#^	4.2 (8.0) ^#^	0.465
	6	5.2 (3.6) ^#^	3.5 (10.7) ^#^	0.640
	12	10.2 (7.0) ^#^	5.6 (9.3) ^#^	0.228
MMO (mm)	3	2.4 (4.0) ^#^	1.4 (5.5) ^#^	0.646
	6	5.0 (4.0) ^#^	3.4 (8.8) ^#^	0.607
	12	10.5 (6.7) ^#^	4.3 (9.0) ^#^	0.098
Protrusive movement (mm)	3	0.3 (−1.3 to 1.0) *	0.0 (−1.0 to 2.5) *	0.829
	6	0.5 (−0.8 to 1.4) *	1.0 (−0.5 to 3.5) *	0.549
	12	0.5 (0 to 2.3) *	0.0 (−2.0 to 2.5) *	0.905
Ipsilateral movement (mm)	3	0.5 (−1.5 to 2.3) *	2.0 (−1.5 to 2.5) *	0.905
	6	0.0 (−1.0 to 0.3) *	1.5 (1.0 to 3.0) *	**0.007**
	12	0.5 (−1.0 to 1.8) *	0.0 (−1.3 to 2.3) *	0.579
Contralateral movement (mm)	3	0.5 (−2.5 to 2.0) *	−0.5 (−2.5 to 1.5) *	0.661
	6	0.2 (2.7) ^#^	0.6 (3.6) ^#^	0.807
	12	1.7 (2.2) ^#^	−0.6 (3.8) ^#^	0.123
MFIQ score	3	−13.7 (20.3) ^#^	−8.8 (24.1) ^#^	0.632
	6	−17.7 (27.3) ^#^	−10.6 (17.7) ^#^	0.501
	12	−37.2 (21.8) ^#^	−11.9 (19.4) ^#^	**0.013**
**Study outcome**	**Follow-up (months)**	**Post-operative scores**		***p***
		Arthroscopy	Arthrocentesis	
Joint noises present, *n* (%)	3	7 (70)	6 (60)	>0.999
	6	7 (70)	8 (80)	>0.999
	12	9 (90)	7 (70)	0.582

Abbreviations: MMO—maximum mouth opening; MFIQ—mandibular function impairment questionnaire; *n*—number of subjects. **^^^**—pre-operative score subtracted from the post-operative score; *—median (first and third quartiles); ^#^—mean (standard deviation). *p*-values in bold indicate statistical significance (≤0.05)

**Table 3:** Linear mixed models and mixed-effects logistic regression results of clinical outcomes.

Outcome	Predictors	Estimates	95% CI	*p*
Pain during mandibular movement (*n* = 20)				
	FU (weeks)	−0.60	−0.80 to −0.40	**<0.001**
	Treatment (ref. = AS)	22.42	5.28 to 39.57	**0.011**
Pain at rest in mm (*n* = 20)				
	FU (weeks)	0.00	−0.11 to 0.12	0.961
	Treatment (ref. = AS)	8.50	−2.68 to 19.68	0.134
MMO without pain in mm (*n* = 20)				
	FU (weeks)	0.13	0.08 to 0.18	**<0.001**
	Treatment (ref. = AS)	0.03	−4.48 to 4.53	0.990
MMO in mm (*n* = 20)				
	FU (weeks)	0.13	0.09 to 0.18	**<0.001**
	Treatment (ref. = AS)	0.63	−3.78 to 5.04	0.775
Protrusive movement in mm (*n* = 20)				
	FU (weeks)	0.02	0.01 to 0.03	**0.006**
	Treatment (ref. = AS)	−0.24	−1.67 to 1.19	0.740
Ipsilateral movement in mm (*n* = 20)				
	FU (weeks)	0.01	−0.01 to 0.04	0.308
	Treatment (ref. = AS)	−1.36	−3.08 to 0.36	0.119
Contralateral movement in mm (*n* = 20)				
	FU (weeks)	0.02	−0.01 to 0.04	0.145
	Treatment (ref. = AS)	−0.14	−1.70 to 1.42	0.861
MFIQ score (*n* = 20)				
	FU (weeks)	−0.39	−0.55 to −0.24	**<0.001**
	Treatment (ref. = AS)	12.94	−0.58 to 26.46	0.060
**Outcome**	**Predictors**	**OR**	**CI**	***p***
Joint noises, present or absent (*n* = 20)				
	FU (weeks)	1.00	1.00 to 1.01	**0.048**
	Treatment (ref. = AS)	0.82	0.09 to 7.40	0.856

Abbreviations: 95% CI—95% Confidence Interval; *n*—number of subjects; FU—Follow-up; ref— reference; AS—arthroscopy; MMO—maximum mouth opening; MFIQ—mandibular function impairment questionnaire. *p*-values in bold indicate statistical significance (≤0.05)

## Data Availability

The raw data supporting the conclusions of this article will be made available by the authors on request.

## Abbreviations

The following abbreviations were used in this manuscript:

TMJ	Temporomandibular joint
MMO	Maximum mouth opening
WMO	Medical Research involving Human Subjects Act
UMCG	University Medical Center Groningen
VAS	Visual Analog Scale
NSAIDs	Non-steroidal anti-inflammatory drugs
CBCT	Cone-beam computed tomography
DJD	Degenerative joint disease
DC/TMD	Diagnostic Criteria for Temporomandibular Dysfunction
ADD	Anterior disc displacement
ADDwR	Anterior disc displacement with reduction
ADDwoR	Anterior disc displacement without reduction
MFIQ	Mandibular function impairment questionnaire
SD	Standard deviation
Q1–Q3	First and third quartiles
CI	Confidence interval
AS	Arthroscopy
OR	Odds ratio
